# Common recorded diagnoses in purebred vs. mixed-breed dogs: a multivariable analysis of primary-care electronic medical records in South Korea

**DOI:** 10.3389/fvets.2026.1925257

**Published:** 2026-08-19

**Authors:** Isaac Yang, Ah-Young Kim, Ji-Su Baek, BokKyung Ku, Hye-Jin Jeong, Kyunghyun Lee

**Affiliations:** 1 College of Veterinary Medicine, Chungbuk National University, Cheongju, Republic of Korea; 2 Animal Disease Diagnostic Division, Animal and Plant Quarantine Agency, Gimcheon, Republic of Korea; 3 Korea Institute for Veterinary Healthcare Policy, Seongnam, Republic of Korea

**Keywords:** adjusted odds ratio, canine disease, electronic medical records, mixed-breed dogs, primary-care practice, purebred dogs, South Korea, veterinary epidemiology

## Abstract

**Introduction:**

In South Korea, the companion-dog population is strongly skewed toward a small number of small-breed purebred dogs, and distinguishing breed-type differences in recorded diagnoses from confounding by age, neuter status, and healthcare utilization is challenging in primary-care data.

**Methods:**

This retrospective study used electronic medical records (EMRs) from primary-care veterinary clinics in South Korea, covering January 2016–December 2024, to compare the adjusted odds of the 20 most frequently recorded diagnoses between purebred and mixed-breed dogs. Records were standardized using Veterinary Nomenclature (VENOM) coding. For each condition, a separate multivariable logistic regression model estimated the adjusted odds ratio (aOR) and 95% confidence interval (CI) for purebred status, adjusting for age, sex, neuter status, and visit frequency. Age was assigned as age at first recorded diagnosis for cases and last observed age for non-cases.

**Results and Discussion:**

After adjustment, purebred dogs showed higher odds of recorded diagnosis for nine conditions, including meningoencephalitis (aOR 2.77; 95% CI 1.17–6.57), intervertebral disc disorder (aOR 1.85; 95% CI 1.49–2.29), myxomatous mitral valve degeneration (aOR 1.57; 95% CI 1.46–1.68), and patellar luxation (aOR 1.54; 95% CI 1.38–1.71). Mammary neoplasm showed higher crude prevalence in mixed-breed dogs, and this association remained statistically significant after adjustment for binary neuter status (aOR 0.80; 95% CI 0.68–0.95; Adj *p* = 0.0202), likely reflecting unmeasured confounding by the timing of neutering. Ten conditions, including chronic kidney disease and unspecified dermatitis, showed no statistically significant difference between groups. These findings indicate breed-type differences in the odds of recorded diagnoses in a Korean primary-care EMR dataset rather than true disease risk or causal effects. Because the cohort was dominated by small-breed dogs and the analyses could not fully account for body size, follow-up duration, detection bias, or diagnostic-coding heterogeneity, the observed associations may reflect breed composition, morphology, owner behavior, healthcare access, and recording practices in addition to underlying biological differences.

## 1 Introduction

The relationship between breed type and disease patterns in dogs has been discussed for decades, often in connection with the idea that greater genetic diversity may influence the distribution of inherited disorders (1, 2). Purebred dogs, shaped by selective breeding for morphological and behavioral traits, can carry higher levels of inbreeding, which may increase homozygosity for some deleterious recessive alleles. Large-scale clinical and genetic studies suggest that this relationship is not uniform across conditions; some disorders are more common in purebred dogs, whereas many others show little or no difference between purebred and mixed-breed populations (2–5).

However, molecular findings alone do not establish how often clinically recorded disorders differ between purebred and mixed-breed dogs in routine veterinary practice.

In clinical datasets, separating breed-type differences from confounding factors is methodologically challenging. In many settings, including South Korea, purebred dogs may receive more frequent veterinary attention, which can increase opportunities for diagnosis and inflate apparent differences in recorded prevalence between groups. Differential neutering practices can further distort crude comparisons for hormone-sensitive conditions such as mammary neoplasia. In addition, the Korean companion-dog population is dominated by a relatively small number of popular toy and small breeds, which complicates interpretation because many common diagnoses are closely linked to body size and morphology.

Several EMR-based studies have examined disorder prevalence by breed type (4, 5). The VetCompass Programme in the United Kingdom reported higher prevalence of some conditions, such as otitis externa and skin masses, in purebred dogs, while many other disorders showed no significant difference (6). A study of 27,254 dogs at the University of California, Davis reported higher prevalence of several inherited disorders in purebred dogs, including intervertebral disc disease, while many other conditions did not differ significantly between purebred and mixed-breed dogs (8, 9). These studies provide important comparative context, but interpretation remains sensitive to age structure, healthcare utilization, and other sources of confounding (10).

The present study used a large multi-clinic Korean primary-care EMR dataset spanning 9 years to estimate breed-type differences in the adjusted odds of the 20 most frequently recorded diagnoses. The analysis used condition-specific age assignment, defining age as the age at first recorded diagnosis for cases and the last observed age for non-cases, and adjusted for sex, neuter status, and visit frequency. The aim was to characterize associations in recorded diagnoses between purebred and mixed-breed dogs in a Korean primary-care context, while explicitly recognizing that EMR-based estimates describe recorded clinical associations rather than causation, true disease incidence, or specific genetic mechanisms.

## 2 Materials and methods

### 2.1 Study design and data source

This was a retrospective observational study based on de-identified EMR data extracted from 103 primary-care veterinary clinics in South Korea, covering the period from January 2016–December 2024 (9 years). The initial data extraction yielded 115,463 raw clinical records from the EMR databases. To maintain the assumption of independent observations and establish a reliable retrospective cohort, deduplication was strictly executed using unique patient identification numbers. For patients with multiple clinic visits, longitudinal tracking was applied, and only the chronological first clinical encounter was retained for baseline characteristic extraction. To ensure temporal consistency and address potential time-varying confounding, demographic variables—specifically including spay/neuter status—were strictly defined based on this initial presentation and treated as fixed baseline characteristics throughout the study. This deduplication process, which also prevented the overrepresentation of frequent visitors, identified a base cohort of 29,354 unique patients.

Subsequently, strict data screening was performed to ensure the integrity of the demographic variables. A total of 536 patients were excluded due to ambiguous or missing entries for sex, neutering status, age, or breed. Additionally, six dogs and their associated 59 records were excluded during data cleaning because of biologically implausible entries (e.g., sex-discordant diagnoses), resulting in a total of 542 excluded patients. The final analytical cohort comprised 28,812 dogs (26,077 purebreds and 2,735 mixed-breeds). Furthermore, to account for potential clustering effects arising from multi-center data collection, the unique clinic identifiers inherent to the original EMR data were utilized. An evaluation of the institutional distribution confirmed a balanced multi-center representation, with the highest patient contribution from any single clinic being only 8.18%. (Supplementary Table 1).

### 2.2 Inclusion criteria and breed classification

All dogs with at least one clinical visit recorded in the contributing clinics during the study period were eligible. Breed type was assigned as purebred or mixed-breed based on the breed information recorded by the attending clinician in the EMR. Specifically, a dog was classified into the mixed-breed group if the recorded breed text entry contained any of the following pre-specified keywords: “Mix,” “Crossbreed,” “Mongrel,” “Batard,” or “Mixed-breed.” Records that did not contain any of these keywords and specified a distinct breed name were classified as purebred. Because breed assignment was based on clinician-recorded and owner-reported EMR entries rather than genetic confirmation, some misclassification may remain, particularly for spelling variants, Korean-language entries, designer crosses, and other ambiguous breed labels.

### 2.3 Diagnostic standardization and disease coding

Diagnostic terms recorded across the contributing clinics were retrospectively reviewed and mapped to standardized Veterinary Nomenclature (VENOM) codes. This mapping was intended to ensure consistent disease classification across heterogeneous clinical coding practices and to allow aggregation of equivalent diagnostic entities across the multi-clinic dataset. To improve data integrity, records with clear charting errors or biologically implausible combinations identified during case-level review were excluded prior to analysis. Because mammary neoplasm can rarely occur in male dogs, sex-discordant mammary-neoplasm records were not excluded automatically on the basis of sex alone; exclusions were limited to records considered clear data-entry errors after review.

### 2.4 Disease selection and prevalence

**Figure 1:**
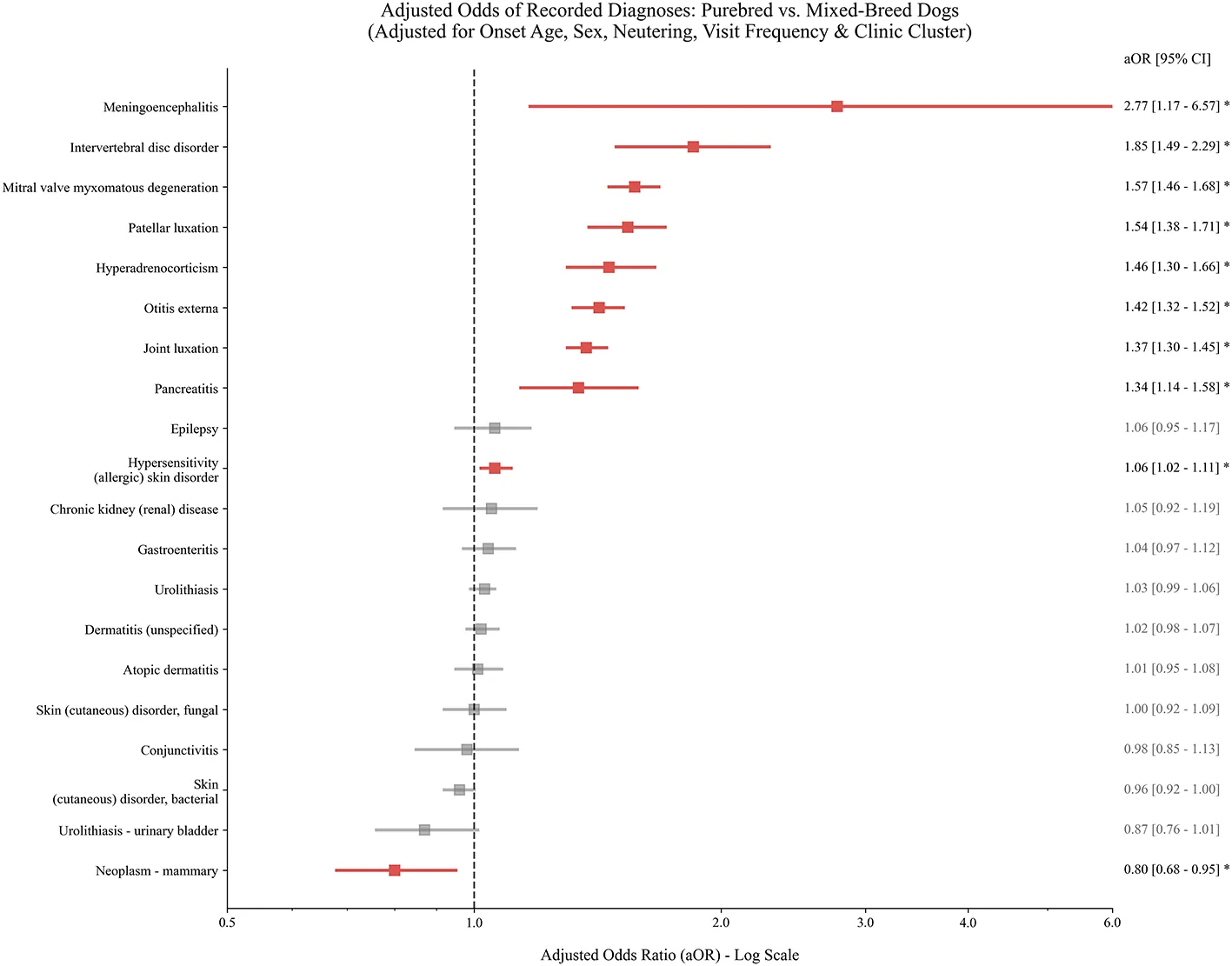
Period prevalence of the top 20 diagnoses, stratified by breed type (purebred vs. mixed-breed). Asterisks indicate statistically significant differences (*p* < 0.05) between groups.

Period prevalence for each condition was calculated as the proportion of unique dogs that received at least one diagnosis of that condition during the observation period. The 20 most prevalent diagnoses across the entire cohort were selected as the conditions of interest. Crude prevalence was stratified by breed type (purebred vs. mixed-breed; Figure 1).

### 2.5 Age-at-onset and last-observed-age handling

A key methodological feature was the individual-level handling of age. Rather than using a single observed age for all dogs, age was assigned for each condition as follows: for diagnosed cases, the age at the first recorded diagnosis of that specific condition; for non-cases, the last observed age (age at the last recorded veterinary visit within the study period). This condition-specific assignment was intended to reduce the age misclassification that can occur when dogs are compared at different life stages for conditions with strong age-of-onset patterns. Although this approach was intended to reduce age misclassification, it does not represent a full time-at-risk framework and may still be influenced by differences in follow-up duration and disease history prior to entry into the EMR dataset.

### 2.6 Statistical analysis

For each of the 20 conditions, a separate binary logistic regression model was fitted with diagnosis status (present/absent) as the outcome and purebred status (purebred vs. mixed-breed) as the primary exposure. Each model adjusted for age, sex, neuter status, and visit frequency. Visit frequency was entered as a continuous variable representing the total number of visits during the observation period and was used as a pragmatic proxy for healthcare utilization. Variance inflation factors (VIFs) were calculated to evaluate potential multicollinearity among the covariates, with a VIF < 5.0 considered acceptable. Each model produced an adjusted odds ratio (aOR) with a 95% confidence interval (CI) for purebred status. Statistical significance was defined as *p* < 0.05. Because visit frequency may itself be influenced by disease occurrence, this covariate was interpreted cautiously in subsequent discussion.

To address potential within-clinic correlation, all logistic regression models were fitted using cluster-robust standard errors, utilizing the clinic identifier as the clustering variable. Missing data in key covariates (age, sex, neuter status) were handled using complete-case analysis. To account for multiple testing across the 20 conditions, *p*-values were adjusted using the Benjamini-Hochberg False Discovery Rate (FDR) procedure. Comprehensive model diagnostics were performed: multicollinearity was assessed using the Variance Inflation Factor (VIF); model stability regarding rare outcomes was verified using the Events Per Variable (EPV) ratio; goodness-of-fit was evaluated via the Hosmer-Lemeshow test; and the linearity of continuous variables was assessed using the Box-Tidwell test. For sensitivity analysis, a Cox proportional hazards model was constructed. All statistical analyses were performed using Python version 3.12.13 (Python Software Foundation) with the pandas (v2.2.2), numpy (v2.0.2), statsmodels (v0.14.6), and lifelines (v0.30.3) libraries. Detailed model diagnostics, multicollinearity assessments, and the complete multivariable logistic regression and Cox proportional hazards sensitivity analysis results are provided in Supplementary Tables 2–7.

### 2.7 Ethics

This study used retrospective, de-identified electronic medical record data and did not involve any prospective intervention on animals. According to institutional policy, formal ethical review and owner consent were not required for analysis of fully anonymized retrospective clinical records.

## 3 Results

### 3.1 Study population

The final cohort comprised 28,812 dogs, representing 113,153 clinical encounters across 103 primary-care clinics in South Korea from 2016 to 2024. At the individual patient level, the mean age of the dogs at their final recorded observation was 8.05 years [standard deviation (SD), 4.52 years; median, 8.05 years; range, 0.0–25.67 years]. Regarding sex distribution, males accounted for 51.2% (14,746/28,812) and females accounted for 48.8% (14,066/28,812) of the population. A high proportion of the cohort was neutered, representing 81.3% (23,413/28,812) of the total patients, while 18.7% (5,399/28,812) remained intact. The population included a diverse array of breeds, heavily dominated by small-breed companion dogs that are highly popular in South Korea. The 10 most frequently recorded breeds were the Maltese (23.8%, *n* = 6,864), Poodle (15.4%, *n* = 4,446), Pomeranian (11.3%, *n* = 3,259), Mixed Breed (9.5%, *n* = 2,735), Bichon Frise (6.3%, *n* = 1,826), Shih Tzu (5.0%, *n* = 1,451), Yorkshire Terrier (4.3%, *n* = 1,236), Chihuahua (3.4%, *n* = 993), Maltipoo (2.7%, *n* = 773), and Dachshund (1.7%, *n* = 499). Together, these top 10 breeds accounted for 83.6% of the entire study population. The mean follow-up duration for the cohort—calculated from the first to the last recorded clinical visit—was 0.24 years (median = 0.00 years), reflecting a predominantly cross-sectional data structure typical of primary-care settings with high proportions of single-visit cases (Supplementary Table 7).

### 3.2 Period prevalence of the top 20 conditions

The 20 most frequently diagnosed conditions and their period prevalence stratified by breed type are shown in Figure 1. Otitis externa was the most prevalent condition overall and had a higher crude prevalence in purebred dogs (13.2%) than in mixed-breed dogs (9.3%); this difference was statistically significant (*p* < 0.05). Patellar luxation showed a similar pattern, with higher crude prevalence in purebred dogs (10.6%) than in mixed-breed dogs (7.2%).

In contrast, mammary neoplasm showed a higher crude period prevalence in mixed-breed dogs (3.6%) than in purebred dogs (2.4%). This difference was examined further in multivariable analyses, particularly with adjustment for neuter status.

Meningoencephalitis, although associated with the highest adjusted odds ratio, showed a modest absolute prevalence of 0.9% in purebred dogs and 0.3% in mixed-breed dogs, illustrating the importance of interpreting adjusted odds alongside crude prevalence.

### 3.3 Adjusted odds ratios for purebred status

**Figure 2:**
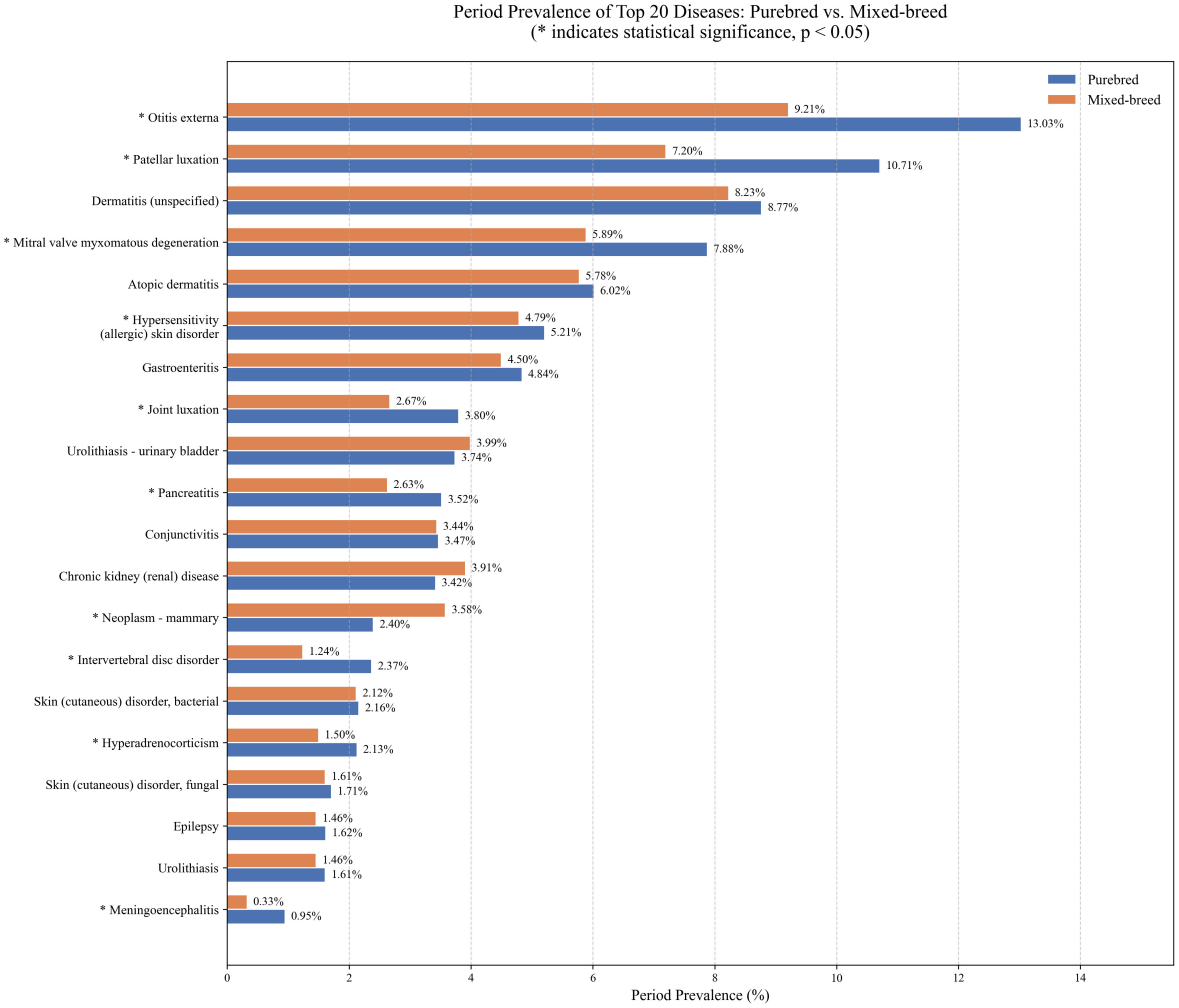
Forest plot of adjusted odds ratios (aOR) for purebred vs. mixed-breed dogs for the top 20 most prevalent conditions. Filled squares indicate statistically significant purebred predisposition (*p* < 0.05); open squares indicate non-significant results. Error bars represent 95% confidence intervals.

After multivariable adjustment for age, sex, neutering status, and clinical visit frequency, nine conditions showed significantly elevated adjusted odds in purebred dogs, while one condition (mammary neoplasm) showed significantly lower adjusted odds (Table 1; Figure 2). Multicollinearity diagnostic testing indicated that the regression models were robust; the variance inflation factors (VIFs) for all included covariates were consistently low (maximum VIF = 1.05), demonstrating the complete absence of multicollinearity among the predictors.

**Table 1:** Adjusted odds ratios (aOR) and 95% confidence intervals (CI) for purebred vs. mixed-breed status for the nine conditions with significantly higher adjusted odds in purebred dogs, after adjustment for age, sex, neuter status, and visit frequency.

Condition	aOR	95% CI	Clinical interpretation
Meningoencephalitis	2.77	1.17–6.57	Higher adjusted odds in purebred dogs
Intervertebral disc disorder	1.85	1.49–2.29	Pattern consistent with small-breed morphology
Myxomatous mitral valve degeneration	1.57	1.46–1.68	Pattern compatible with breed-related cardiac susceptibility
Patellar luxation	1.54	1.38–1.71	Pattern consistent with small-breed skeletal morphology
Hyperadrenocorticism	1.46	1.30–1.66	Pattern compatible with breed-associated endocrine susceptibility
Otitis externa	1.42	1.32–1.52	Pattern compatible with ear morphology and dermatologic traits
Joint luxation	1.37	1.30–1.45	Higher adjusted odds in purebred dogs
Pancreatitis	1.34	1.14–1.58	Pattern compatible with breed-associated metabolic susceptibility
Hypersensitivity (allergic) skin disorder	1.06	1.02–1.11	Slightly higher adjusted odds in purebred dogs

Ten conditions showed non-significant adjusted odds ratios (aOR range 0.80–1.06), including common dermatological conditions (unspecified dermatitis, atopic dermatitis, fungal and bacterial skin disorders), gastroenteritis, chronic kidney disease, epilepsy, and urolithiasis (Figure 1).

### 3.4 Conditions with significantly higher adjusted odds in purebred dogs

**Meningoencephalitis (aOR 2.77, 95% CI 1.17–6.57)**. The largest adjusted odds ratio for purebred status was observed for meningoencephalitis (Figure 2).

**Intervertebral disc disorder (aOR 1.85, 95% CI 1.49–2.29)**. Purebred dogs showed significantly higher adjusted odds of recorded diagnosis for intervertebral disc disorder (Figure 2).

**Myxomatous mitral valve degeneration (aOR 1.57, 95% CI 1.46–1.68)**. Purebred dogs showed significantly higher adjusted odds of recorded diagnosis for myxomatous mitral valve degeneration (Figure 2).

**Patellar luxation (aOR 1.54, 95% CI 1.38–1.71)**. Purebred dogs showed significantly higher adjusted odds of recorded diagnosis for patellar luxation (Figure 2).

**Hyperadrenocorticism (aOR 1.46, 95% CI 1.30–1.66)**. Purebred dogs showed significantly higher adjusted odds of recorded diagnosis for hyperadrenocorticism (Figure 2).

**Otitis externa (aOR 1.42, 95% CI 1.32–1.52)**. Purebred dogs showed significantly higher adjusted odds of recorded diagnosis for otitis externa (Figure 2).

**Joint luxation (aOR 1.37, 95% CI 1.30–1.45)**. Purebred dogs showed significantly higher adjusted odds of recorded diagnosis for joint luxation (Figure 2).

**Pancreatitis (aOR 1.34, 95% CI 1.14–1.58)**. Purebred dogs showed significantly higher adjusted odds of recorded diagnosis for pancreatitis (Figure 2).

**Hypersensitivity (allergic) skin disorder (aOR 1.06, 95% CI 1.02–1.11)**. Purebred dogs showed slightly but significantly higher adjusted odds of recorded diagnosis for hypersensitivity skin disorders (Figure 2).

### 3.5 Conditions without significant breed-type differences

Ten of the top 20 conditions showed non-significant adjusted odds, including unspecified dermatitis (aOR 1.02, 95% CI 0.98–1.07), atopic dermatitis (aOR 1.01, 95% CI 0.95–1.08), chronic kidney disease (aOR 1.05, 95% CI 0.92–1.19), gastroenteritis (aOR 1.04, 95% CI 0.97–1.12), epilepsy (aOR 1.06, 95% CI 0.95–1.17), and urolithiasis (aOR 1.03, 95% CI 0.99–1.06). These results are consistent with broader literature reporting that many common disorders, particularly those with multifactorial or environmental causes, are similarly distributed across breed types.

### 3.6 Mammary neoplasm and confounding by neutering status

Mammary neoplasm showed a higher crude period prevalence in mixed-breed dogs (3.6%) than in purebred dogs (2.4%). After multivariable adjustment including binary neuter status, the association remained statistically significant (aOR 0.80; 95% CI 0.68–0.95; Adj *p* = 0.0202). However, this finding illustrates how unmeasured differences in management practices (e.g., timing of neutering) can resemble breed-type differences when crude comparisons are interpreted without full temporal adjustment. Because there is no established genetic mechanism that would favor higher mammary-tumor risk in mixed-breed dogs, this pattern is more readily explained by confounding. Neutering, particularly before the first or second oestrous cycle, substantially reduces mammary-tumor risk, and a higher proportion of intact females in the mixed-breed group would be expected to raise their crude prevalence.

## 4 Discussion

### 4.1 Breed-type differences for structural, neurological, and endocrine conditions

In this East Asian primary-care cohort, purebred dogs had higher adjusted odds of recorded diagnosis for several structural, neurological, cardiac, and endocrine conditions. The largest associations were observed for meningoencephalitis, intervertebral disc disorder, myxomatous mitral valve degeneration, and patellar luxation, all of which are conditions previously linked to particular breed groups, body conformations, or inherited susceptibilities. Because the Korean purebred population in this dataset was heavily dominated by toy and small breeds, these findings should be interpreted in light of breed composition and morphology as well as possible underlying biological factors (1–3).

The magnitude of the association for meningoencephalitis is notable, but it should be interpreted cautiously because the absolute prevalence was low and the result may be strongly influenced by the distribution of specific toy breeds within the cohort. More broadly, the present data identify associations in recorded diagnoses and do not directly demonstrate a genetic mechanism or establish hybrid vigor (11). Previous studies have reported overrepresentation of toy and small purebred breeds in disorders such as meningoencephalitis and patellar luxation, and chondrodystrophic breeds in intervertebral disc disorder. These prior findings provide context for the patterns observed here, but the present study did not directly test genetic mechanisms.

### 4.2 Detection bias and visit-frequency adjustment

A major challenge in comparing recorded diagnoses between purebred and mixed-breed dogs is differential healthcare utilization. Dogs that visit clinics more frequently have more opportunities for incidental, earlier, or more complete diagnosis, which can inflate apparent differences between groups. Visit frequency was therefore included as a covariate to partially account for this source of bias.

However, visit frequency is an imperfect proxy for detection bias. It does not capture within-visit diagnostic intensity, owner decision-making, or access to care, and disease itself may increase subsequent visit frequency, creating the possibility of overadjustment or collider bias. The estimates should therefore be interpreted with caution, and future analyses would benefit from using pre-diagnosis visit frequency or time-to-event approaches when feasible (8, 9).

### 4.3 Mammary neoplasm as an example of confounding

The mammary neoplasm finding provides a useful illustration of why crude breed-type comparisons can be misleading. Crude prevalence was higher in mixed-breed dogs, and while this difference remained significant after adjusting for binary neuter status, it indicates that unmeasured management factors likely contributed substantially to the observed pattern. Because mammary-tumor occurrence is strongly influenced by reproductive status and timing of neutering, this result is more appropriately interpreted as an example of confounding than as evidence of a breed-type biological effect (6).

### 4.4 Conditions without clear breed-type differences

Ten of the 20 most frequently recorded conditions did not show statistically significant differences in adjusted odds between purebred and mixed-breed dogs. This is itself informative, because it suggests that many common disorders in primary-care practice may be similarly distributed across breed types or may be more strongly influenced by environmental, management, or multifactorial causes than by breed type alone. These findings are broadly consistent with earlier studies in which many common disorders showed no significant purebred vs. mixed-breed difference (3, 4).

### 4.5 Comparison with previous studies

The present findings are broadly consistent with previous veterinary epidemiology studies while also reflecting the distinctive composition of the Korean companion-dog population. The higher adjusted odds of otitis externa, intervertebral disc disorder, and patellar luxation in purebred dogs are concordant with earlier primary-care studies from the United Kingdom and the University of California, Davis (4, 7–9, 11). At the same time, interpretation in the Korean setting requires particular caution because a large proportion of the cohort consisted of popular toy and small breeds, and many of the observed associations are compatible with body-size and morphology effects as much as with breed-type categories themselves (4, 10).

The main contribution of this study is therefore not to establish a single explanation for purebred vs. mixed-breed differences, but to document how such differences appear in a large Korean primary-care EMR dataset after multivariable adjustment. The study also highlights the importance of age handling, neuter-status adjustment, and cautious interpretation of healthcare-utilization measures in breed-type comparisons.

### 4.6 Limitations

Several limitations should be considered when interpreting these findings. First, breed type was based on owner-reported and clinician-recorded EMR entries rather than genetic testing, so some degree of misclassification is possible. This issue may be particularly relevant for spelling variants, Korean-language entries, designer crosses, and dogs with ambiguous breed labels.

Second, the study population was heavily dominated by small and toy breeds, and bodyweight or size information was not incorporated into the primary models. As a result, some observed breed-type differences may partly reflect morphology and size rather than breed type *per se*.

Third, the retrospective multi-clinic design introduces potential variability in diagnostic thresholds, coding practices, and case ascertainment across clinics. Because clinic-level clustering was not the main focus of the present analysis, residual within-clinic correlation may remain.

Fourth, age was handled using age at first recorded diagnosis for cases and last observed age for non-cases. Although this approach was intended to reduce age misclassification, it is not equivalent to a full time-at-risk framework and may still be biased by differences in follow-up duration or diagnosis prior to entry into the EMR dataset.

Fifth, visit frequency was used as a proxy for healthcare utilization, but this variable may be influenced by the outcome itself and therefore may not fully resolve detection bias. Sixth, important covariates such as bodyweight, geographic region, diet, comorbidities, and detailed reproductive history, including timing of neutering, were unavailable or incomplete. Finally, this observational design estimates associations in recorded diagnoses and cannot establish causation or identify a specific genetic mechanism.

## 5 Conclusion

In this multi-clinic Korean primary-care EMR study, purebred and mixed-breed dogs differed in the odds of several common recorded diagnoses after adjustment for age, sex, neuter status, and visit frequency. Purebred dogs showed higher adjusted odds of recorded diagnosis for several conditions, including meningoencephalitis, intervertebral disc disorder, myxomatous mitral valve degeneration, and patellar luxation, whereas many other common conditions did not differ significantly by breed type.

These findings should be interpreted as associations observed in a retrospective primary-care EMR dataset rather than as estimates of true disease risk or evidence of a specific genetic mechanism. The study population was heavily dominated by small and toy breeds, and the available data did not fully capture body size, follow-up time, timing of neuter status, or pre-diagnosis healthcare utilization. Accordingly, the observed differences may reflect breed composition, morphology, owner behavior, healthcare access, and diagnostic or coding practices, in addition to any underlying biological factors.

Within these limitations, the study shows that routinely collected EMR data can identify clinically relevant differences in recorded diagnosis patterns across breed types in Korean dogs. Future studies using clearer breed classification, bodyweight or size variables, clinic-level clustering methods, and time-to-event approaches will be important for clarifying the mechanisms underlying these associations.

## Data Availability

The original contributions presented in the study are included in the article/Supplementary material, further inquiries can be directed to the corresponding author/s.

## References

[1] Nicholas FW Wade CM Williamson P. Hybrid vigor in dogs? Vet J. (2016) 214:77–83. doi: 10.1016/j.tvjl.2016.05.013 27387730

[2] DonnerJ AndersonH DavisonS HughesAM BouirmaneJ LindqvistJ . Frequency and distribution of 152 genetic disease variants in over 100,000 mixed breed and purebred dogs. PLoS Genet. (2018) 14:e1007361. doi: 10.1371/journal.pgen.100736129708978 PMC5945203

[3] ForsythKK AkeyJM KaeberleinM PromislowDEL CreevyKE. Dog aging project consortium. lifetime prevalence of owner-reported medical conditions in the 25 most popular dog breeds in the dog aging project pack. Front Vet Sci. (2023) 10:1140417. doi: 10.3389/fvets.2023.114041738026653 PMC10655140

[4] Bellumori TP Famula TR Bannasch DL Belanger JM Oberbauer AM. Prevalence of inherited disorders among mixed-breed and purebred dogs: 27,254 cases (1995-2010). J Am Vet Med Assoc. (2013) 242:1549–55. doi: 10.2460/javma.242.11.1549 23683021

[5] OberbauerAM BelangerJM BellumoriTP BannaschDL FamulaTR. Ten inherited disorders in purebred dogs by functional breed groupings. Canine Genet Epidemiol. (2015) 2:9. doi: 10.1186/s40575-015-0021-x26401337 PMC4579393

[6] Dorn CR Taylor DON Schneider R Hibbard HH Klauber MR. Factors influencing canine mammary cancer development and postsurgical survival. J Natl Cancer Inst. (1968) 43:1249–61. 4319248

[7] BrownEA DickinsonPJ MansourT SturgesBK AguilarM YoungAE . FGF4 retrogene on CFA12 is responsible for chondrodystrophy and intervertebral disc disease in dogs. Proc Natl Acad Sci U S A. (2017) 114:11476–81. doi: 10.1073/pnas.170908211429073074 PMC5664524

[8] O'NeillDG ChurchDB McGreevyPD ThomsonPC BrodbeltDC. Prevalence of disorders recorded in dogs attending primary-care veterinary practices in England. PLoS ONE. (2014) 9:e90501. doi: 10.1371/journal.pone.009050124594665 PMC3942437

[9] O'NeillDG JamesH BrodbeltDC ChurchDB PegramC. Prevalence of commonly diagnosed disorders in UK dogs under primary veterinary care: results and applications. BMC Vet Res. (2021) 17:69. doi: 10.1186/s12917-021-02775-333593363 PMC7888168

[10] ChoiY O'NeillDG IvesE BrownTH LopesBA AlvesLC . Meningoencephalitis of unknown origin in dogs under veterinary referral care in England (2017-2021): a multicenter case-control study. Front Vet Sci. (2025) 12:1710593. doi: 10.3389/fvets.2025.171059341383969 PMC12690937

[11] O'NeillDG MeesonRL SheridanA ChurchDB BrodbeltDC. The epidemiology of patellar luxation in dogs attending primary-care veterinary practices in England. Canine Genet Epidemiol. (2016) 3:4. doi: 10.1186/s40575-016-0034-027280025 PMC4898461

