# Supplementary material for: Common recorded diagnoses in purebred vs. mixed-breed dogs: a multivariable analysis of primary-care electronic medical records in South Korea
Source: Front Vet Sci. 2026 Aug 19;13:1925257. doi: 10.3389/fvets.2026.1925257 (PMC13533715; doi:10.3389/fvets.2026.1925257)
Supplement: Supplementary file 1 [file Table_1.docx]

**Supplementary Table 1. Distribution of the final analytical cohort across the top 10 contributing primary-care veterinary clinics.**

| Rank | Clinic Identifier (Masked) | Final Patient Count (n) | Percentage (%) |
| --- | --- | --- | --- |
| 1 | Clinic A | 2357 | 8.18 |
| 2 | Clinic B | 2345 | 8.14 |
| 3 | Clinic C | 2300 | 7.98 |
| 4 | Clinic D | 1838 | 6.38 |
| 5 | Clinic E | 1414 | 4.91 |
| 6 | Clinic F | 839 | 2.91 |
| 7 | Clinic G | 826 | 2.87 |
| 8 | Clinic H | 808 | 2.8 |
| 9 | Clinic I | 786 | 2.73 |
| 10 | Clinic J | 681 | 2.36 |

**Supplementary Table 2. Variance inflation factor (VIF) for the independent variables included in the multivariable logistic regression models.**

VIF values used to assess multicollinearity among the covariates (purebred status, age at last observation, visit frequency, sex, and neuter status). A VIF value < 5.0 indicates the absence of problematic multicollinearity across all models.

| **Variable** | **VIF** |
| --- | --- |
| is_purebred | 1 |
| last_observed_age | 1.03 |
| visit_count | 1.01 |
| sex_M | 1.04 |
| is_neutered | 1.05 |

**Supplementary Table 3. Model diagnostics evaluating rare outcome risk and goodness-of-fit for the top 20 most prevalent conditions.**

Events per variable (EPV) were calculated to assess model stability, with an EPV > 10 indicating a low risk of rare outcome bias. The Hosmer-Lemeshow goodness-of-fit test evaluates model calibration; p-values are reported with the caveat of the known hyper-sensitivity of this test in large cohorts (N > 29,000).

| **Disease** | **Total_Events** | **Events_per_Variable(EPV)** | **Rare_Outcome_Risk** | **Hosmer-Lemeshow_p-value** |
| --- | --- | --- | --- | --- |
| Patellar luxation | 2990 | 598 | No | 0.013 |
| Otitis externa | 3649 | 729.8 | No | 0 |
| Mitral valve myxomatous degeneration | 2217 | 443.4 | No | 0 |
| Dermatitis (unspecified) | 2513 | 502.6 | No | 0 |
| Atopic dermatitis | 1727 | 345.4 | No | 0 |
| Chronic kidney (renal) disease | 998 | 199.6 | No | 0.1293 |
| Epilepsy | 463 | 92.6 | No | 0 |
| Intervertebral disc disorder | 651 | 130.2 | No | 0.0509 |
| Hypersensitivity (allergic) skin disorder | 1490 | 298 | No | 0 |
| Skin (cutaneous) disorder, fungal | 489 | 97.8 | No | 0.0074 |
| Joint luxation | 1065 | 213 | No | 0.1809 |
| Hyperadrenocorticism | 596 | 119.2 | No | 0 |
| Pancreatitis | 990 | 198 | No | 0.0043 |
| Gastroenteritis | 1386 | 277.2 | No | 0.0011 |
| Skin (cutaneous) disorder, bacterial | 622 | 124.4 | No | 0.0046 |
| Meningoencephalitis | 258 | 51.6 | No | 0.0342 |
| Urolithiasis - urinary bladder | 1083 | 216.6 | No | 0 |
| Neoplasm - mammary | 723 | 144.6 | No | 0 |
| Conjunctivitis | 998 | 199.6 | No | 0.0103 |
| Urolithiasis | 460 | 92 | No | 0 |

**Supplementary Table 4. Case counts, crude prevalence, and multivariable logistic regression results for the 20 most frequently recorded diagnoses in purebred versus mixed-breed dogs.**

Adjusted odds ratios (aOR), 95% confidence intervals (CI), and raw p-values were derived from models adjusting for age, sex, neuter status, and visit frequency, utilizing cluster-robust standard errors to account for clinic-level clustering. Adjusted p-values (FDR) were calculated using the Benjamini-Hochberg procedure to correct for multiple testing across the 20 conditions.

| **Disease** | **Total_Cases** | **Purebred_Cases** | **Mixed_Cases** | **Purebred_Crude_Prev(%)** | **Mixed_Crude_Prev(%)** | **Adjusted_OR** | **95%_CI** | **Raw_p-value** | **Adj_p-value(FDR)** |
| --- | --- | --- | --- | --- | --- | --- | --- | --- | --- |
| Patellar luxation | 2990 | 2793 | 197 | 10.71 | 7.2 | 1.54 | [1.38 - 1.71] | 0 | 0 |
| Otitis externa | 3649 | 3397 | 252 | 13.03 | 9.21 | 1.42 | [1.32 - 1.52] | 0 | 0 |
| Mitral valve myxomatous degeneration | 2217 | 2056 | 161 | 7.88 | 5.89 | 1.57 | [1.46 - 1.68] | 0 | 0 |
| Dermatitis (unspecified) | 2513 | 2288 | 225 | 8.77 | 8.23 | 1.02 | [0.98 - 1.07] | 0.3698 | 0.4623 |
| Atopic dermatitis | 1727 | 1569 | 158 | 6.02 | 5.78 | 1.01 | [0.95 - 1.08] | 0.7155 | 0.7892 |
| Chronic kidney (renal) disease | 998 | 891 | 107 | 3.42 | 3.91 | 1.05 | [0.92 - 1.19] | 0.4988 | 0.5868 |
| Epilepsy | 463 | 423 | 40 | 1.62 | 1.46 | 1.06 | [0.95 - 1.17] | 0.2817 | 0.3913 |
| Intervertebral disc disorder | 651 | 617 | 34 | 2.37 | 1.24 | 1.85 | [1.49 - 2.29] | 0 | 0 |
| Hypersensitivity (allergic) skin disorder | 1490 | 1359 | 131 | 5.21 | 4.79 | 1.06 | [1.02 - 1.11] | 0.0053 | 0.0131 |
| Skin (cutaneous) disorder, fungal | 489 | 445 | 44 | 1.71 | 1.61 | 1 | [0.92 - 1.09] | 0.9624 | 0.9624 |
| Joint luxation | 1065 | 992 | 73 | 3.8 | 2.67 | 1.37 | [1.3 - 1.45] | 0 | 0 |
| Hyperadrenocorticism | 596 | 555 | 41 | 2.13 | 1.5 | 1.46 | [1.3 - 1.66] | 0 | 0 |
| Pancreatitis | 990 | 918 | 72 | 3.52 | 2.63 | 1.34 | [1.14 - 1.58] | 0.0005 | 0.0015 |
| Gastroenteritis | 1386 | 1263 | 123 | 4.84 | 4.5 | 1.04 | [0.97 - 1.12] | 0.2935 | 0.3913 |
| Skin (cutaneous) disorder, bacterial | 622 | 564 | 58 | 2.16 | 2.12 | 0.96 | [0.92 - 1.0] | 0.0473 | 0.086 |
| Meningoencephalitis | 258 | 249 | 9 | 0.95 | 0.33 | 2.77 | [1.17 - 6.57] | 0.0204 | 0.0409 |
| Urolithiasis - urinary bladder | 1083 | 974 | 109 | 3.74 | 3.99 | 0.87 | [0.76 - 1.01] | 0.0612 | 0.102 |
| Neoplasm - mammary | 723 | 625 | 98 | 2.4 | 3.58 | 0.8 | [0.68 - 0.95] | 0.0091 | 0.0202 |
| Conjunctivitis | 998 | 904 | 94 | 3.47 | 3.44 | 0.98 | [0.85 - 1.13] | 0.7497 | 0.7892 |
| Urolithiasis | 460 | 420 | 40 | 1.61 | 1.46 | 1.03 | [0.99 - 1.06] | 0.1244 | 0.1913 |

**Supplementary Table 5. Box-Tidwell linearity check results for continuous covariates (age and visit frequency) across the 20 diagnosis models.**

P-values represent the significance of the interaction term between the continuous variable and its natural logarithm. While p-values < 0.05 formally indicate a deviation from strict log-linearity, this reflects the high statistical power and hyper-sensitivity of the test in large sample sizes rather than severe model assumption violations.

| **Disease** | **Age_Linearity_p** | **Visit_Linearity_p** |
| --- | --- | --- |
| Patellar luxation | 0.0001 | 0.0002 |
| Otitis externa | 0 | 0 |
| Mitral valve myxomatous degeneration | 0 | 0 |
| Dermatitis (unspecified) | 0 | 0 |
| Atopic dermatitis | 0 | 0 |
| Chronic kidney (renal) disease | 0.0023 | 0 |
| Epilepsy | 0.8753 | 0 |
| Intervertebral disc disorder | 0 | 0 |
| Hypersensitivity (allergic) skin disorder | 0 | 0 |
| Skin (cutaneous) disorder, fungal | 0.0122 | 0 |
| Joint luxation | 0.5214 | 0 |
| Hyperadrenocorticism | 0 | 0 |
| Pancreatitis | 0.0103 | 0 |
| Gastroenteritis | 0.631 | 0 |
| Skin (cutaneous) disorder, bacterial | 0.0015 | 0 |
| Meningoencephalitis | 0 | 0 |
| Urolithiasis - urinary bladder | 0 | 0 |
| Neoplasm - mammary | 0 | 0.0127 |
| Conjunctivitis | 0.2858 | 0 |
| Urolithiasis | 0 | 0 |

**Supplementary Table 6. Sensitivity analysis using a Cox proportional hazards model to estimate hazard ratios (HR) for the 20 most frequently recorded diagnoses in purebred versus mixed-breed dogs.**

This table presents the results of a time-to-event sensitivity analysis. Hazard ratios (HR), 95% confidence intervals (CI), and p-values are reported for the purebred group compared to the mixed-breed reference group. The models were adjusted for sex, neuter status, and visit frequency, utilizing the age at the first recorded diagnosis as the event time and the age at the last observed visit as the censoring time. Empty rows (e.g., for Epilepsy, Meningoencephalitis) indicate conditions for which the Cox proportional hazards model failed to converge.

| **Disease** | **Hazard_Ratio(HR)** | **HR_95%_CI** | **Cox_p-value** |
| --- | --- | --- | --- |
| Patellar luxation | 1.52 | [1.31 - 1.76] | 0 |
| Otitis externa | 1.5 | [1.32 - 1.71] | 0 |
| Mitral valve myxomatous degeneration | 1.7 | [1.45 - 2.0] | 0 |
| Dermatitis (unspecified) | 1.12 | [0.98 - 1.29] | 0.1052 |
| Atopic dermatitis | 1.11 | [0.94 - 1.3] | 0.2244 |
| Chronic kidney (renal) disease | 1.29 | [1.06 - 1.58] | 0.0124 |
| Epilepsy |  |  |  |
| Intervertebral disc disorder | 2.12 | [1.5 - 3.0] | 0 |
| Hypersensitivity (allergic) skin disorder | 1.16 | [0.97 - 1.39] | 0.0976 |
| Skin (cutaneous) disorder, fungal |  |  |  |
| Joint luxation | 1.42 | [1.12 - 1.81] | 0.0037 |
| Hyperadrenocorticism | 1.64 | [1.19 - 2.26] | 0.0023 |
| Pancreatitis | 4.86 | [3.23 - 7.32] | 0 |
| Gastroenteritis | 1.15 | [0.95 - 1.38] | 0.1513 |
| Skin (cutaneous) disorder, bacterial | 0.38 | [0.26 - 0.55] | 0 |
| Meningoencephalitis |  |  |  |
| Urolithiasis - urinary bladder | 0.96 | [0.78 - 1.17] | 0.6635 |
| Neoplasm - mammary | 0.96 | [0.77 - 1.19] | 0.7074 |
| Conjunctivitis | 1.1 | [0.89 - 1.36] | 0.3927 |
| Urolithiasis |  |  |  |

**Supplementary Table 7. Summary statistics of the follow-up duration (in years) for the study cohort.**

Follow-up duration was calculated as the time between the first and last recorded clinical visits for each dog (N = 28,812). The median follow-up time of 0 years reflects the predominantly cross-sectional nature of the primary-care electronic medical record (EMR) dataset, comprising a high proportion of single-visit or transient cases.

|  | **followup_years** |
| --- | --- |
| **count** | 28,812 |
| **mean** | 0.24 |
| **std** | 0.58 |
| **min** | 0 |
| **25%** | 0 |
| **50%** | 0 |
| **75%** | 0.12 |
| **max** | 7.98 |
